# Raucherassoziierte Malignome: Mehr als nur das Lungenkarzinom

**DOI:** 10.1007/s00117-022-00992-x

**Published:** 2022-04-01

**Authors:** Katharina Martini

**Affiliations:** Institut für Diagnostische und Interventionelle Radiologie, Universitätsspital Zürich, Rämistr. 100, 8091 Zürich, Schweiz

**Keywords:** Tabak, Rauchen, Neoplasien, Karzinogenese, Nikotin, Tobacco, Smoking, Neoplasms, Carcinogenesis, Nicotine

## Abstract

**Hintergrund:**

Tabakkonsum ist die häufigste vermeidbare Ursache für Krebserkrankungen und Krebstodesfälle. Tabakkonsum steht nicht nur im Zusammenhang mit Lungenkrebs, sondern hat Einfluss auf die Krebsentstehung in fast allen Organsystemen.

**Ziel der Arbeit:**

Das Ziel dieses Übersichtsartikels ist es, auf die verschiedenen beteiligten Organsysteme in der tabakassoziierten Krebsentstehung näher einzugehen.

**Material und Methoden:**

Zunächst erfolgt eine kurze Einführung in die Thematik, gefolgt von einer ausführlichen Beschreibung der verschiedenen Tumorentitäten, die mit dem Tabakkonsum assoziiert sind.

**Ergebnisse:**

Der Tabakkonsum wird mit der Verursachung vieler Krebsarten in Verbindung gebracht. Nach aktuellen Erkenntnissen kann Tabakkonsum Mund‑, Pharynx‑, Larynx‑, Ösophagus‑, Magen‑, Nieren‑, Pankreas‑, Leber‑, Blasen‑, Zervix‑, Kolon- und Rektumkarzinome sowie die akute myeloische Leukämie verursachen.

**Diskussion:**

Tabakkonsum ist nicht nur Hauptursache für die Entstehung von Lungenkrebs, sondern hat auch einen großen Einfluss auf die Entstehung bösartiger Erkrankungen in anderen Organsystemen. Daher muss bei der Bildauswertung von Rauchern ein besonderes Augenmerk auf andere maligne Begleiterkrankungen gelegt werden.

## Abstract (en)

**Background:**

Tobacco use is the leading preventable cause of cancer and cancer deaths. Tobacco use is not only related to lung cancer, but has an impact on a wide range of different cancer entities in almost every organ system.

**Objectives:**

The aim of this review article is to shed light on the different organ systems involved in tobacco-associated carcinogenesis.

**Materials and methods:**

For this purpose, first a brief introduction into the topic is given, followed by a detailed description of the different tumor entities associated with tobacco use.

**Results:**

Tobacco consumption has been clearly implicated in the causation of many types of cancer, affecting multiple organ systems. Based on current evidence, tobacco use can cause cancer of the mouth and throat, larynx, esophagus, stomach, kidney, pancreas, liver, bladder, cervix, colon and rectum, and acute myeloid leukemia.

**Conclusion:**

Tobacco use is not only main cause in the development of lung cancer, but has a major impact in the development of cancer in other organ systems. Therefore, special attention must be given to possible concomitant malignancies when evaluating images of smokers.

Der Tabakkonsum ist ein etablierter Risikofaktor für die Karzinogenese in fast allen Organsystemen [18]. Tabak verursacht Neoplasien der Lunge, des Larynx und Pharynx, der Mundhöhle, des Ösophagus und des Magens, des Urogenitaltrakts, der Leber und des Pankreas sowie des Dickdarms und des hämatologischen Systems [21]. Je nach Lokalisation und Geschlecht sind 8–82 % dieser Neoplasien auf das Rauchen zurückzuführen [22]. Das Ziel dieses Übersichtsartikels ist es, die verschiedenen beteiligten Organsysteme in der tabakassoziierten Krebsentstehung aufzuzeigen.

## Mund, Nasennebenhöhlen, Pharynx und Larynx

**Figure 1:**
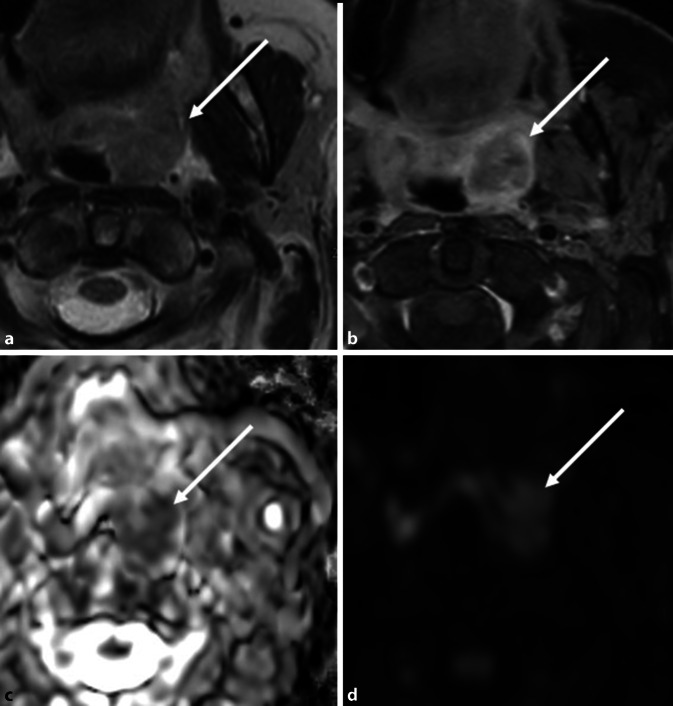

Diese Organgruppe ist neben der Lunge das am häufigsten betroffene System der tabakassoziierten Karzinogenese [15]. Anfang der 1990er Jahre des vorigen Jahrhunderts analysierte eine Studie 454 Patienten mit Mundhöhlenkarzinom und stellte fest, dass 60 % der Patienten mit Mundhöhlenkarzinom rauchten [17]. Über 95 % der Neubildungen waren Plattenepithelkarzinome (Abb. 1; [17]).

Folgestudien zeigten, dass Tabak oxidativen Stress auf das Gewebe auslöst, eine epigenetische Veränderung oraler Epithelzellen verursachen sowie mehrere systemische Immunfunktionen hemmen kann und so die Entstehung von Neoplasien begünstigt [12, 15].

Primäre Zigarren- und Pfeifenraucher haben ein etwas geringeres Risiko für Lungenkrebs als Zigarettenraucher, aber ihr Risiko für Larynx‑, Pharynx‑, Mundhöhlen- und Ösophagusneoplasien ist ähnlich, wenn nicht sogar größer als das von Zigarettenrauchern [1]. Orale Tabakprodukte, wie z. B. Schnupftabak oder Kautabak, haben eine Assoziation mit Neubildungen der Wangenschleimhaut, des Zahnfleischs und der inneren Oberfläche der Lippen [1]. Zudem kann die Kombination von Tabakkonsum mit Alkohol das Krebsrisiko vervielfachen [19].

## Ösophagus und Magen

Tabakrauchen erhöht das Risiko für ein Plattenepithelkarzinom des Ösophagus stark und das Risiko für ein Adenokarzinom des Ösophagus moderat [25]. Die Raucherentwöhnung verringert zeitabhängig das Risiko für ein Plattenepithelkarzinom des Ösophagus, während es einen begrenzten Einfluss auf das Risiko eines Adenokarzinoms des Ösophagus hat [25].

Auf ähnliche Weise ist das Rauchen mit der Entstehung von Magenkrebs vergesellschaftet, wobei Fundus und Kardia bzw. proximale, nahe dem Ösophagus gelegene Magenanteile häufiger betroffen sind als weiter distal gelegene [20]. Auch bei Ösophagus- und Magenkarzinomen stellt der Genuss von Alkohol ein additives Krebsentstehungsrisiko dar [19].

## Pankreas und Leber

### Pankreas

**Figure 2:**
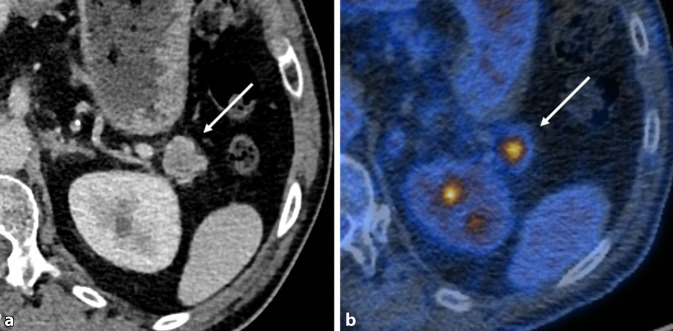

Rauchen ist einer der wichtigsten Risikofaktoren für die Entstehung des Pankreaskarzinoms (Abb. 2): Das Risiko, an einem Pankreaskarzinom zu erkranken, ist bei Rauchern etwa doppelt so hoch wie bei Personen, die nie geraucht haben. Es wird angenommen, dass etwa 25 % der Pankreaskarzinome durch Zigarettenrauchen verursacht werden. Auch das Rauchen von Zigarren und die Verwendung von rauchlosen Tabakprodukten erhöhen das Risiko. Das Risiko für die Entstehung eines Pankreaskarzinom sinkt jedoch, sobald eine Person mit dem Rauchen aufhört [11].

### Leber

Rauchen führt zur Entstehung von Stoffen mit kanzerogenem Potenzial, die das Risiko eines hepatozellulären Karzinoms (HCC) erhöhen. Zudem hat das Rauchen einen additiven kanzerogenen Effekt bei Patienten mit Virushepatitis [4]. Tabakrauchen wird mit der Unterdrückung von Tumorsuppressorgenen, wie beispielsweise p53, sowie der T‑Zell-Antwort in Verbindung gebracht [4]. Zudem erhöht das Rauchen den Eisengehalt der Leber und favorisiert somit die Entwicklung einer Leberfibrose und damit das Risiko der Entstehung eines HCC [5].

## Nieren und Blase

**Figure 3:**
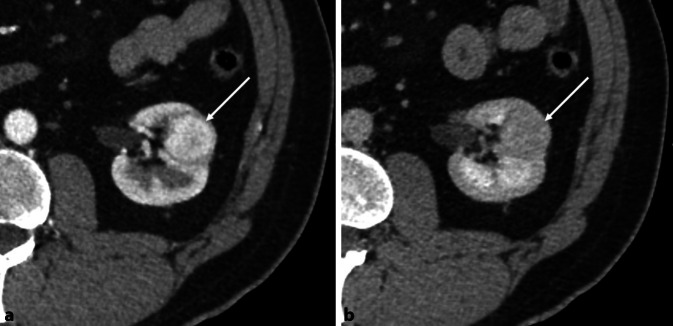

Mehreren Studien deuten bei Rauchern auf ein deutlich erhöhtes Risiko für die Entstehung von Neoplasien des Urogenitaltrakts hin (Abb. 3; [28]). Zigarettenraucher haben ein etwa dreifach höheres Risiko für Harnwegskrebs als Nichtraucher [28]: In Europa kann etwa die Hälfte der Fälle von Harnwegskrebs bei Männern und ein Drittel der Fälle bei Frauen dem Rauchen zugeschrieben werden [28].

### Blase

Der am besten untersuchte Zusammenhang von Tabakrauchen und der Krebsentstehung im Urogenitalsystem ist jener der Harnblase und geht bis auf die 50er Jahre des 20. Jahrhunderts zurück: Diesen Untersuchungen zufolge ist das Rauchen sowohl bei Männern als auch bei Frauen der am besten etablierte Risikofaktor für die Entstehung von Blasenkrebs [10]. Das Risiko von Rauchern, an Blasenkrebs zu erkranken, erhöhte sich gemäß dieser Studien um den Faktor 3 im Vergleich zu Nichtrauchern [10]. Die Zusammensetzung von Zigaretten hat sich jedoch in den letzten 50 Jahren verändert, die Verringerung der Teer- und Nikotinkonzentrationen [26] gingen mit einem Anstieg der Konzentration spezifischer Karzinogene, darunter β‑Naphthylamin, ein bekanntes Blasenkarzinogen, und tabakspezifische Nitrosamine, einher [9]. Mit diesen Veränderungen in den Bestandteilen des Zigarettenrauchs haben epidemiologische Studien ein höheres rauchassoziiertes relatives Risiko für Lungenkrebs beobachtet [9]. Ein kürzlich erschienener Bericht der New England Bladder Study, einer großen bevölkerungsbasierten Fall-Kontroll-Studie, deutet darauf hin, dass die Assoziation zwischen Zigarettenrauchen und Blasenkrebs möglicherweise ebenfalls zugenommen hat [2, 7].

### Niere

Auch die Niere bleibt von den Auswirkungen des Tabakkonsums nicht verschont: In einer Studie von Yuan et al. [27] in welcher 1024 Patienten mit Nierenzellkarzinom (RCC) eingeschlossen wurden, hatten Raucher, die 40 oder mehr Zigaretten/Tag rauchten, im Vergleich zu lebenslangen Nichtrauchern ein fast zweifach erhöhtes RCC-Risiko [27]. Es gab hingegen keine messbaren Unterschiede im RCC-Risiko zwischen Rauchern von gefilterten und nicht gefilterten Zigaretten oder zwischen denen, die Zigarettenrauch tief und leicht inhalierten [27]. Starke Zigarrenraucher (14 oder mehr Zigarren/Woche) zeigten mit 70 % eine statistisch signifikante Erhöhung des Risikos, an einem RCC zu erkranken. Beim Konsum von Pfeifen oder rauchlosem Tabak wurde hingegen kein erhöhtes RCC-Risiko beobachtet [27]. Im Vergleich zu aktiven Rauchern erfuhren diejenigen, die vor 10 oder mehr Jahren mit dem Rauchen aufgehört hatten, eine statistisch signifikante Reduzierung des RCC-Risikos um 30 % [27].

## Ovarien und Zervix

### Ovar

Bei Frauen in der westlichen Welt ist das Ovarialkarzinom die sechsthäufigste diagnostizierte Krebsart und die sechsthäufigste Krebstodesursache [13]. Das Ovarialkarzinom ist der tödlichste gynäkologische Tumor mit einer 5‑Jahres-Gesamtüberlebensrate von 30–40 % [13]. Eine wachsende Zahl von Studien zeigt das Zigarettenrauchen als potenziellen Risikofaktor für die Entstehung des Ovarialkazinoms [14]. Die stärkste Assoziation scheint mit muzinösen Ovarialtumoren zu bestehen, während die Assoziation mit anderen histologischen Typen weniger gesichert ist [14].

### Zervix

**Figure 4:**
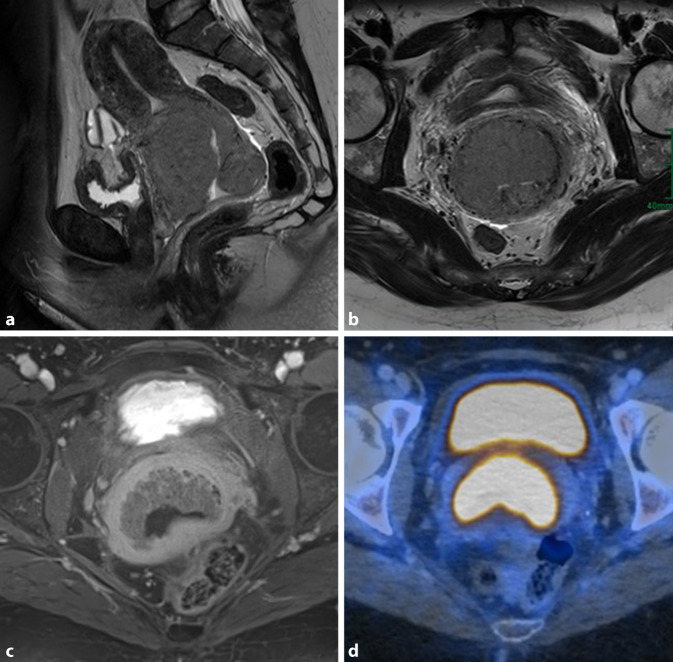

Frauen, die rauchen, haben ein etwa doppelt so hohes Risiko, am Zervixkarzinom zu erkranken, wie diejenigen, die nicht rauchen [6]. Mehrere Faktoren scheinen Einfluss auf die zervikale Karzinogenese zu haben, insbesondere durch direkte lokale karzinogene Wirkung und lokale Immunsuppression [6]: Tabaknebenprodukte wurden im Zervixschleim von Raucherinnen gefunden. Forscher glauben, dass diese Substanzen die DNA von Gebärmutterhalszellen schädigen und zur Entstehung von Gebärmutterhalskrebs beitragen können [6]. Zudem vervielfacht das Rauchen das kanzerogene Risiko bei einer Infektion mit dem humanen Papillomvirus (HPV; Abb. 4).

## Kolon und Rektum

Dickdarmkrebs (kolorektales Karzinom, CRC) ist weltweit die dritthäufigste Krebsart und die zweithäufigste krebsbedingte Todesursache [3]. Wie bei anderen multifaktoriellen Erkrankungen ist die Entwicklung von CRC das Ergebnis eines komplexen Zusammenspiels zwischen Lebensstil und genetischen Faktoren [8]. Das Rauchen von Zigaretten wird mit der Entstehung von adenomatösen Polypen in Verbindung gebracht [24]. Eine bevölkerungsbezogene Fall-Kontroll-Studie zu Dickdarmkrebs beobachtete sowohl bei Männern als auch bei Frauen eine etwa 50 %ige Erhöhung des CRC-Risikos durch das Rauchen von mehr als einer Packung Zigaretten pro Tag. Diejenigen, die mit dem Rauchen aufgehört hatten, blieben einem erhöhten Risiko ausgesetzt, selbst wenn sie vor über 10 Jahren damit aufgehört haben [24]. Die Ergebnisse dieser Studie deuten darauf hin, dass die gerauchte Menge an Zigaretten ein wichtigerer Faktor sein könnte als die Gesamtzahl der gerauchten Jahre. Interessanterweise war weder das Rauchen von Zigarren noch von Pfeifen mit einem erhöhten Darmkrebsrisiko verbunden [24].

## Akute myeloische Leukämie

Die akute myeloische Leukämie (AML) ist eine bösartige hämatologische Erkrankung, gekennzeichnet durch eine blockierte Differenzierung, maligne Proliferation und Apoptose normaler hämatopoetischer Zellen [16]. AML hat multifaktorielle Risikofaktoren, wie Umweltfaktoren, genetische Faktoren, Alter und Ethnie. Zudem zeigten Shi et al. [23] in einer Metaanalyse von 20 Fall-Kontroll-Studien, mit insgesamt 7538 AML-Patienten und 137.924 gesunden Kontrollpersonen, dass die Assoziation zwischen Zigarettenrauchen und der Entstehung einer AML insbesondere in der kaukasischen Bevölkerung erhöht ist. Ferner wurde gezeigt, dass Rauchen während der Schwangerschaft das Risiko der Entstehung einer AML im Kindesalter erhöht [23].

## Fazit für die Praxis

- Tabakkonsum ist nicht nur Hauptursache für die Entstehung von Lungenkrebs, sondern hat auch einen großen Einfluss auf die Entstehung bösartiger Erkrankungen in anderen Organsystemen.
- Als Radiologen sollten wir die Prädilektionsstellen der tabakassoziierten Karzinogenese kennen und bei Personen dieser Risikogruppe ein besonderes Augenmerk auf diese Organsysteme legen, um etwaige Zufallsbefunde nicht zu übersehen.
- Das Erkennen des Tabakkonsums als Risikofaktor für die Entstehung verschiedener Tumorentitäten kann einen positiven Einfluss auf die Prävention von solchen Erkrankungen und damit auch auf die öffentliche Gesundheit haben.
